# Transcatheter Aortic Valve Implantation by Intercostal Access: Initial Experience with a No-Touch Technique

**DOI:** 10.3390/jcm12165211

**Published:** 2023-08-10

**Authors:** Nina Sophie Pommert, Xiling Zhang, Thomas Puehler, Hatim Seoudy, Katharina Huenges, Jan Schoettler, Assad Haneya, Christine Friedrich, Janarthanan Sathananthan, Stephanie L. Sellers, David Meier, Oliver J. Mueller, Mohammed Saad, Derk Frank, Georg Lutter

**Affiliations:** 1 Department of Cardiac and Vascular Surgery, University Medical Center Schleswig-Holstein, Campus Kiel, 24105 Kiel, Germany; thomas.puehler@uksh.de (T.P.); katharina.huenges@uksh.de (K.H.); jan.schoettler@uksh.de (J.S.); assad.haneya@uksh.de (A.H.); christine.friedrich@uksh.de (C.F.); 2 DZHK—German Centre for Cardiovascular Research, Partner Site Hamburg/Kiel/Lübeck, 24105 Kiel, Germany; derk.frank@uksh.de; 3 Department of Internal Medicine III (Cardiology, Angiology, and Critical Care), University Medical Center Schleswig-Holstein, Campus Kiel, 24105 Kiel, Germany; hatim.seoudy@uksh.de (H.S.); oliver.mueller@uksh.de (O.J.M.); mohammed.saad@uksh.de (M.S.); 4 Cardiovascular Translational Laboratory, Providence Research & UBC Centre for Heart Lung Innovation, Vancouver, BC V6Z 1Y6, Canada; jsathananthan@providencehealth.bc.ca (J.S.); ssellers@providencehealth.bc.ca (S.L.S.); david.meier1291@gmail.com (D.M.); 5 Centre for Heart Valve Innovation and Division of Cardiology, St. Paul’s Hospital, University of British Columbia, Vancouver, BC V6T 1Z4, Canada; 6 UBC Centre for Cardiovascular Innovation, Vancouver, BC V6Z 1Y6, Canada

**Keywords:** TAVI, transaortic TAVI, aortic valve replacement, minimal invasive surgery, intercostal

## Abstract

Background: Transcatheter aortic valve implantation (TAVI) is now a well-established therapeutic option in an elderly high-risk patient cohort with aortic valve disease. Although most commonly performed via a transfemoral route, alternative approaches for TAVI are constantly being improved. Instead of the classical mini-sternotomy, it is possible to achieve a transaortic access via a right anterior mini-thoracotomy in the second intercostal space. We describe our experience with this sternum- and rib-sparing technique in comparison to the classical transaortic approach. Methods: Our retrospective study includes 173 patients who were treated in our institution between January 2017 and April 2020 with transaortic TAVI via either upper mini-sternotomy or intercostal thoracotomy. The primary endpoint was 30-day mortality, and secondary endpoints were defined as major postoperative complications that included admission to the intensive care unit and overall hospital stay, according to the Valve Academic Research Consortium 3. Results: Eighty-two patients were treated with TAo-TAVI by upper mini-sternotomy, while 91 patients received the intercostal approach. Both groups were comparable in age (mean age: 82 years) and in the proportion of female patients. The intercostal group had a higher rate of peripheral artery disease (41% vs. 22%, *p* = 0.008) and coronary artery disease (71% vs. 40%, *p* < 0.001) with a history of percutaneous coronary intervention or coronary artery bypass grafting, resulting in significantly higher preinterventional risk evaluation (EuroScore II 8% in the intercostal vs. 4% in the TAo group, *p* = 0.005). Successful device implantation and a reduction of the transvalvular gradient were achieved in all cases with a significantly lower rate of trace to mild paravalvular leakage in the intercostal group (12% vs. 33%, *p* < 0.001). The intercostal group required significantly fewer blood transfusions (0 vs. 2 units, *p* = 0.001) and tended to require less reoperation (7% vs. 15%, *p* = 0.084). Hospital stays (9 vs. 12 d, *p* = 0.011) were also shorter in the intercostal group. Short- and long-term survival in the follow-up showed comparable results between the two approaches (30-day, 6-month- and 2-year mortality: 7%, 23% and 36% in the intercostal vs. 9%, 26% and 33% in the TAo group) with acute kidney injury (AKI) and reintubation being independent risk factors for mortality. Conclusions: Transaortic TAVI via an intercostal access offers a safe and effective treatment of aortic valve stenosis.

## 1. Introduction

The transfemoral approach is widely accepted as a safe and easy access through which to perform transcatheter aortic valve implantation (TAVI) [1,2,3]. If peripheral artery cannulation is impossible due to calcification, tortuosity or size, surgical approaches are needed. A transaortic route via an upper J- or T-mini-sternotomy (TAo-TAVI) represents a valuable alternative to the established transapical access, which is proven to pose a risk of bleeding, ventricular damage and myocardial infarction [4,5,6,7,8,9]. Therefore, the TAo-TAVI access has been proposed by several surgical groups as a potential first-line option for surgical TAVI [10].

Based on positive experiences with minimally invasive surgical aortic valve replacement (SAVR), a modification of the TAo-TAVI approach has been established to avoid any touching of the ribs or sternum—the intercostal transaortic TAVI [11]. Avoiding sternotomy in SAVR lowered bleeding risk and increased chest wall stability, favoring accelerated recovery after surgery [12]. Encouraging results using this technique for TAVI have been described in single center studies, including a technical note from our group [13,14].

In this study, we analyze our experience with intercostal TAVI and compare the results to those obtained with TAo-TAVI via upper mini-sternotomy at our institution.

## 2. Materials and Methods

### 2.1. Study Design

The study was approved by our local Ethics Review Board (ERB) (D529/16), and patients’ informed consent was obtained.

From January 2017 to April 2020, a total of 173 patients with severe aortic stenosis who had undergone TAVI by transaortic access at our institution were identified retrospectively. Discussions with all potential TAVI candidates were held beforehand with a multidisciplinary heart team, and the decision to employ either TAVI or SAVR was made based on current guidelines [15].

Three-dimensional reconstructive computed tomography (CT) of the aorta including the groin vessels was performed in each case to evaluate the feasibility of a peripheral vascular access. Echocardiography was carried out to define the patient’s aortic valve morphology, and was combined with aortic root CT to assess the size of the prosthetic heart valve. Coronary angiography was used to exclude any coronary artery disease requiring surgical revascularization.

Following current guidelines, a transfemoral approach was favored. Throughout the study period, 673 patients received transfemoral TAVI. For this procedure, a minimal inner diameter of the common femoral and iliac artery of 7 mm was required. Where transfemoral access was unsuitable due to severe peripheral vessel stenosis, excessive tortuosity or calcifications of the femoral or iliac arteries, patients were treated with a transaortic approach. In cases of extensive calcification of the ascending aorta, transaortic access was rejected in favor of a transapical procedure (102 transapical TAVIs in the study period). The intercostal approach required a maximum distance of 80 mm between the skin and aorta, and a minimum distance of 65 mm from the puncture point to the annular plane for optimal valve positioning. Due to the risk of injury during sternotomy, a TAo approach was avoided after coronary artery bypass grafting. All patients treated with TAo- or intercostal TAVI were included in the analysis.

### 2.2. Procedural Details

The TAVI procedures were performed in a hybrid operating theater. Unlike in transfemoral TAVI, where patients are usually awake and only slightly sedated, general anesthesia and mechanical ventilation were required for the transaortic access. Between the two transaortic groups, the anesthesia protocol was identical.

Temporary pacemaker wires were implanted for rapid pacing during stent release, and for protection against arrhythmias in the early perioperative period. A pigtail catheter was inserted into the femoral artery to assist hemodynamic monitoring and valve positioning.

**Figure 1:**
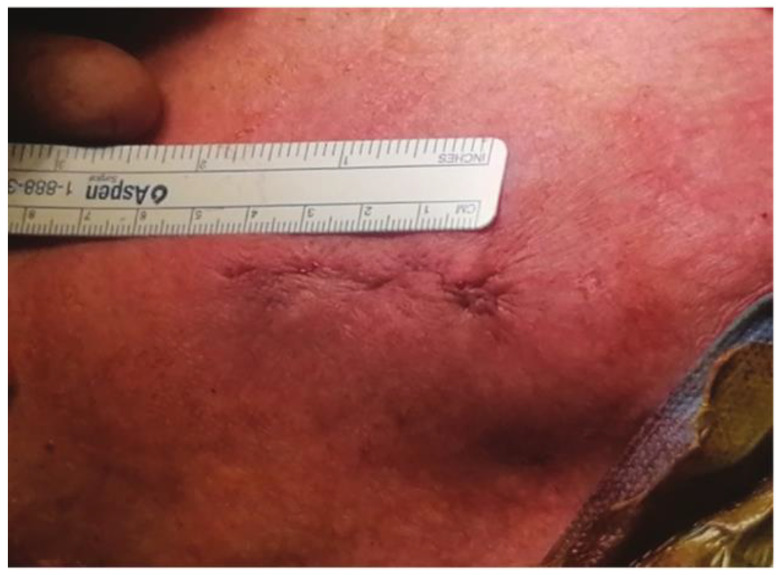
4–5 cm skin incision.

**Figure 2:**
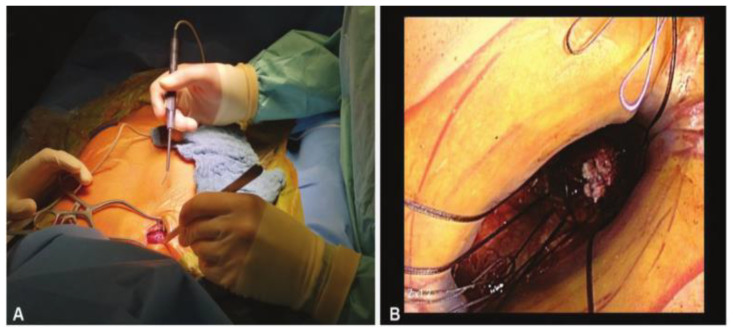
(**A**) Minimally invasive approach through a 1.5-inch skin incision located in the second right intercostal space; (**B**) Only one finger space for knotting.

For the intercostal approach, surgical access was obtained through a right anterior mini-thoracotomy using a 4–5 cm skin incision (Figure 1) placed in the second intercostal space. A soft tissue retractor was inserted for optimal exposure (Figure 2). The approach allows for the avoidance of any cartilage transection, rib dislocation or sternal touch, as well as leaving the right internal mammary artery, vein, and the parietal pleura untouched. The upper third of the pericardium was opened along the outer curvature of the aorta. Two felt-pledget pursue-string sutures were placed on the aorta, and a 6F-introducer sheath was inserted using the Seldinger technique. The aortic valve was passed with a stiff pre-shaped guidewire. After achieving a stable wire position, an 18-F sheath was inserted into the aorta. By using this access, it was possible to implant every available valved stent. Controlled TAVI was then performed under fluoroscopy and under a rapid pacing of 120–180/min. Transesophageal echocardiography and fluoroscopy were routinely performed to verify correct prosthesis guidance, deployment and positioning.

Protamine was given, a chest tube was inserted into the right pleura, and the small incision was closed in standard fashion.

The procedural details regarding TAo-TAVI using upper mini-sternotomy have previously been described [16].

The perioperative complications were observed, including death, cerebrovascular events, perioperative myocardial infarction, permanent pacemaker implantation, and paravalvular leakage (PVL). All patients were followed up after the operation in our TAVI registry. Post-TAVI echocardiography outcomes were assessed by transthoracic echocardiography during postprocedural inpatient treatment.

### 2.3. Statistical Analysis

The outcomes and endpoints for the two groups were determined according to the Valve Academic Research Consortium 3 (VARC3) criteria. The endpoints assessed included: (1) Procedure length; (2) Length of stay on the intensive care unit (ICU); (3) Hospitalization; (4) Major postoperative complications (i.e., blood transfusion ≥ 2; PVL, etc.); (5) 30-day mortality.

The categorical variables were expressed as absolute numbers and percentages, and the continuous variables were expressed as means with standard deviation means, or medians with interquartile ranges. Comparisons between the TAo and intercostal groups were made using the $x^{2}$ test or Fisher’s exact test for categorical variables, and the *t*-test or Wilcoxon rank sum test for continuous variables. Kaplan–Meier curves were used to analyze the difference in mortality between the two groups. Cox regression was used to analyze the risk factors affecting mortality. All statistical analyses were performed using SPSS version 28.0 (IBP Corp., Armonk, NY, USA), with a two-sided *p*-valve of <0.05 considered statistically significant.

## 3. Results

All patients were followed up during hospitalization and after discharge for up to two years. Within the study period of three years, a total of 81 patients underwent TAo, and 92 patients underwent intercostal TAVI.

Baseline characteristics are presented in Table 1. The mean ages of the intercostal group and TAo group were 82 ± 5 years and 82 ± 6 years (*p* = 0.284), respectively. The body mass index (BMI) was significantly higher in the TAo group with 25.9 kg/m^2^ (interquartile range [IQR]: 23.2 to 30.6), compared to 24.2 kg/m^2^ (IQR: 24.2 to 27.4) in the intercostal group (*p* = 0.022). Dyslipidemia was more common in the intercostal group at an incidence of 69%, and 33% in the TAo group, respectively (*p* < 0.001). Patients in the intercostal group showed a higher incidence of peripheral artery disease at 41% compared to 22% in the TAo group (*p* = 0.008). Moreover, coronary artery disease was more frequent in the intercostal group, as there were more patients with a history of percutaneous coronary intervention (PCI) than in the TAo group. The intercostal group also featured a significantly higher rate of prior cardiac surgery (19% vs. 2%, *p* = 0.001), including mainly coronary artery bypass grafting. Overall, the estimated mortality risk after cardiac surgery was significantly higher in the intercostal group, which exhibited a Euroscore I of 22.9% and EuroScore II of 8.0% versus 14.9% and 4.1% in the TAo group (*p* < 0.001 and *p* = 0.005).

**Figure 3:**
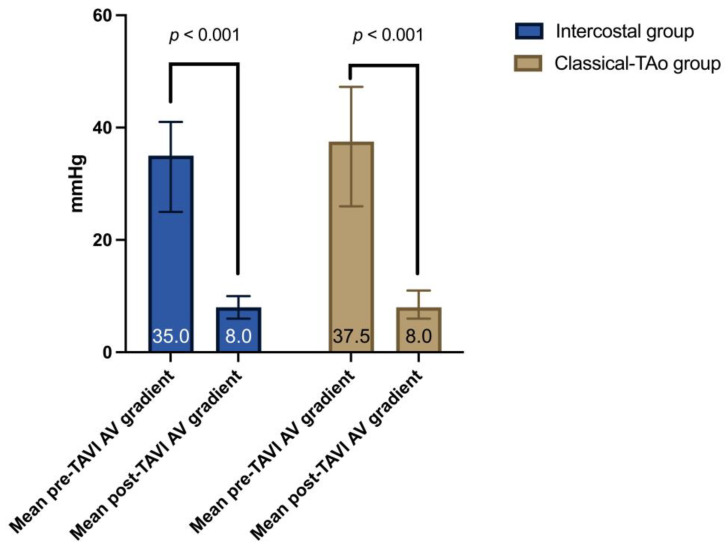
Comparison of preoperative and postoperative mean AV gradient difference.

Preoperative and postoperative echocardiographic characteristics are shown in Table 2. There were no significant differences in echocardiographic characteristics between the two groups. As expected, the mean aortic valve gradient was significantly lower in both groups after the intervention (Figure 3).

Comparing the implanted valve models, the balloon-expandable Edwards Sapien 3 or 3 Ultra were used in 47 patients (52%) in the intercostal group and in 14 patients (17%) in TAo group. The majority of the TAo group were implanted with the self-expanding CoreValve Evolut R or Pro (68 patients, 83%), compared to 44 patients (48%) in the intercostal group (*p* < 0.001).

Procedural outcomes are presented in Table 3. Intercostal patients tended to have shorter procedure durations and ICU stays, but there was no significance in the analyzed cohort. However, the length of hospitalization in the intercostal group was significantly lower than in the TAo group (9 days, IQR: 8–13 vs. 12 days, IQR: 8–18.25, *p* = 0.011). Patients in the TAo group required significantly more postoperative blood transfusions than patients in the intercostal group with 2 Units (IQR: 0–4) and 0 Units (IQR: 0–1), respectively (*p* = 0.001). They also were more likely to experience PVL after the implantation (33% in the TAo vs. 12% in the intercostal group, *p* < 0.001).

Eighteen patients required rethoracotomy, including 6 (7%) in the intercostal group and 12 (15%) patients in the TAo group (*p* = 0.084). The reasons for rethoracotomy were bleeding (5% in both groups) and wound healing disorders, the latter tending to be more frequent after TAo-TAVI (1% in the intercostal vs. 7% in the TAo group, *p* = 0.054). Notably, two patients in the TAo group required surgical aortic valve replacement due to dislocation of the prosthesis. The occurrence of high-grade atrioventricular blocks requiring pacemaker implantation were comparable between the two groups (10% in the intercostal vs. 12% in the TAo group, *p* = 0.628).

More patients in the intercostal group required reintubation (11% vs. 7%, *p* = 0.392), but this was not significant for our analysis. The reasons for the reintubation besides rethoracotomy for bleeding were pneumonia and respiratory exhaustion.

**Figure 4:**
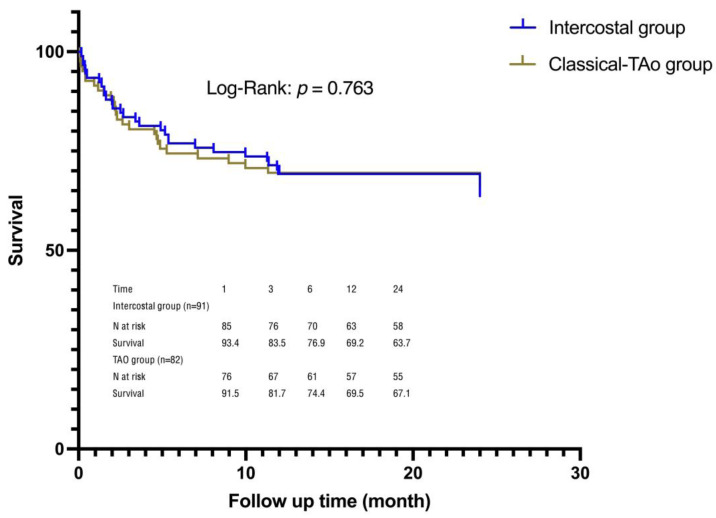
Kaplan–Meier survival analysis curves for two study groups.

The Kaplan–Meier curve demonstrates the difference in mortality between the two groups (Figure 4). There were thirteen deaths within the 30-day follow-up period. Six occurred in the intercostal group due to pneumonia, sepsis and multi-organ failure (5 cases) and pericardial tamponade. The other seven in the TAo group occurred due to cardiovascular reasons. There was no significant difference in the 30-day all-cause mortality between the two groups (*p* = 0.364). Acute kidney injury (AKI) and reintubation were independent risk factors for mortality after Cox multi-factor regression analysis (Table 4). The remaining factors were not significant.

## 4. Discussion

The current study summarizes our experience using intercostal transaortic *TAVI* for 91 patients with severe symptomatic aortic valve stenosis that were not candidates for surgical aortic valve replacement or transfemoral TAVI. The key findings are as follows: (1) Patients treated by the intercostal TAo approach were less likely to suffer trace to mild PVL than TAo patients; (2) Blood transfusion was more likely to be required for TAo cases; (3) The length of hospitalization in the intercostal group was significantly lower than that in the TAo group; (4) AKI and reintubation were independent risk factors for mortality.

Taking into consideration the relevant literature combined with our institutional experience related to the TAo approach, the intercostal TAo approach for TAVI is currently the first choice in our department when the transfemoral approach is unsuitable. The intercostal approach requires a distance of less than 80 mm from the skin above the intercostal space towards the aorta, and more than 65 mm from the puncture point to the annular plane to ensure the safe positioning and deployment of the valved stents. All available valved stents were implanted via this route.

As one of the serious complications after TAVI, paravalvular leakage seriously affects patients’ immediate survival and long-term prognoses. Studies have shown that even mild PVL will reduce the survival rate of TAVI patients [2,17]. We observed that the intercostal TAo group was less likely to suffer trace to mild PVL in the postoperative echocardiography. This may be related to a lower depth of implantation of the valved stent. In addition, most patients in the TAo-TAVI group received self-expandable valves, which exert less radial force than their balloon-expandable counterparts, and therefore pose a risk for PVL [18]. For patients with calculable surgical risk, minimally invasive surgical aortic valve replacement via an intercostal approach represents a valuable alternative to avoid paravalvular leakage [19]. Lamelas et al. demonstrated excellent results (30-day mortality 2.2%) for minimally invasive right thoracotomy aortic valve surgery, especially in their subgroup of patients over 80 years, which is comparable to our cohort (STS 4% vs. 6% in our intercostal group) [20].

The TAo approach is performed with an upper mini-sternotomy and a pericardiotomy to expose the ascending aorta. This increases the potential for bleeding and collateral injury. Indeed, in the present study, sternal revision was the main reason for re-thoracotomy in the TAo group. This also explains why patients treated by the TAo approach required more blood transfusions. Interestingly, the procedure duration, and length of ICU stay should theoretically be shorter for the intercostal TAVI, but these variables did not differ between the two groups. This might be due to the fact that intercostal TAVI is still a new technique, and more experience is necessary. Encouragingly, we observed shorter hospital stays in the intercostal TAo group. As the surgeons’ team gains experience, the duration of the procedure decreases, the length of hospitalization will be shorter, and the risk of nosocomial infection will be lower. Moreover, patients who underwent intercostal TAo-TAVI at our institution had a significantly higher preoperative surgical risk compared to those who underwent TAo-TAVI. However, the frequency of postoperative complications, as well as short- and long-term survival were comparable between the two groups. Post-TAVI aortic valve gradients were significantly lower in both groups. This indicates the efficacy and safety of intercostal TAo-TAVI.

We found that AKI was an independent risk factor for 2-year mortality. The contrast agents used in TAVI and the hypotension produced by rapid pacing may affect kidney function and lead to acute kidney injury.

In previous studies, 30-day and 1-year mortality rates were as high as 44.4% and 55.6% in patients who developed AKI after TAVI [21,22]. AKI may be a marker of multi-organ failure, and is therefore associated with higher mortality. Studies have shown that contrast media volume over 100 mL was associated with higher contrast-induced nephropathy and mortality after PCI [23]. Therefore, it is important to reduce the amount of contrast agent used during implantation. The use of echocardiography or magnetic resonance imaging guidance during deployment may reduce the amount of contrast agent used for TAVI [24,25].

Furthermore, we observed that reintubation was also an independent risk factor for mortality. In the present study, the main reason for reintubation was the need for a rethoracotomy due to bleeding. Optimization of the procedure and coagulation management are key features for the future treatment.

Indeed, the only and main exclusion criteria for the intercostal TAVI are calcification of the distal ascending aorta in the area of procedural entrance, and in a minority, the innominate artery, the proximal arch or the right subclavian artery.

## 5. Limitations

This study is a single-center investigation with inherent limitations and potential bias in reporting clinical and echocardiographic findings. The group sizes in this first experience study are relatively small. Propensity matching was not performed, leading to differences in baseline characteristics and valve type between the groups, which may have affected the results. Any increase in operator experience over time may improve the results. Further multicenter analysis and long-term follow-up are necessary.

## 6. Conclusions

Compared to the classical approach for transaortic TAVI by upper mini-sternotomy, the intercostal access in a no-touch technique demonstrates a comparably low rate of perioperative complications in a significantly higher risk patient cohort. In this first experience German study, patients receiving the intercostal access showed less paravalvular leakage and tended to require less reoperation. This resulted in a significantly lower need for blood transfusions and shorter hospital stays. Based on our research, we rate the intercostal access as a safe and effective approach to performing transaortic TAVI in a high-risk patient cohort.

## Figures and Tables

**Table 1:** Baseline Characteristics.

Characteristics	Intercostal Group (*n* = 91)	TAo Group (*n* = 82)	*p* Value
Age, years, Mean ± SD	82.2 ± 5.1	81.7 ± 5.8	0.524
Female	48 (52.7%)	40 (48.8%)	0.602
STS PROM,% Mean ± SD	6.1 (4.1–8.4) 6.6 ± 3.4	4.0 (3.2–6.5) 5.1 ± 3.5	0.277
Log Euroscore I, % Mean ± SD	22.9 (16.6–31.9) 25.3 ± 13.3	14.9 (9.6–21.4) 16.6 ± 8.5	**<0.001**
Log Euroscore II, % Mean ± SD	8.0 (4.1–10.0) 8.7 ± 6.37	4.1 (2.8–6.4) 5.3 ± 5.1	**0.005**
Body mass index, kg/m^2^ Mean ± SD	24.2 (21.4–27.4) 25.2 ± 5.1	25.9 (23.3–30.6) 27.0 ± 5.6	**0.022**
NYHA III/IV	82 (90.1%)	72 (87.8%)	0.628
Dyslipidemia	63 (69.2%)	27 (32.9%)	**<0.001**
Hypertension	85 (93.4%)	70 (85.4%)	0.084
COPD	24 (26.4%)	18 (22.0%)	0.498
Diabetes	32 (35.2%)	26 (31.7%)	0.631
Atrial fibrillation	50 (54.9%)	40 (48.8%)	0.418
Coronary artery disease	65 (71.4%)	33 (40.2%)	<0.001
Prior myocardial infarction	45 (49.5%)	31 (37.8%)	0.304
Prior PCI	34 (37.4%)	16 (19.5%)	**0.010**
Prior cardiac surgery	22 (24.2%)	2 (2.4%)	**<0.001**
Prior cerebrovascular disease	23 (25.3%)	19 (23.2%)	0.747
Peripheral vascular disease	37 (40.7%)	18 (22.0%)	**0.008**

COPD = chronic obstructive pulmonary disease; NYHA = New York Heart Association; PCI = percutaneous coronary intervention; SD = standard deviation; STS PROM = The Society of Thoracic Surgeons Predicted Risk of Mortality.

**Table 2:** Echocardiographic Data.

Echocardiographic Characteristics	Intercostal Group (*n* = 91)	TAo Group (*n* = 82)	*p* Value
Pre-TAVI LVEF, %	55 (45–60)	55 (45–60)	0.800
Mean pre-TAVI AV gradient, mmHg Mean ± SD	35 (25–41) 34.7 ± 13.1	37.5 (26–47.2) 40.2 ± 18.9	0.229
Post-TAVI LVEF, %	55 (50–60)	60 (46–65)	0.395
Mean post-TAVI AV gradient, mmHg Mean ± SD	8 (6–10) 8.8 ± 3.9	8 (6–11) 9.2 ± 5.3	0.900

AV = aortic valve; LVEF = left ventricular ejection fraction; SD = standard deviation; TAVI = transcatheter aortic valve replacement.

**Table 3:** Outcomes.

Outcomes	Intercostal Group (*n* = 91)	TAo Group (*n* = 82)	*p* Value
Device success	90 (100%)	82 (100%)	1.000
Self-expandable valves	44 (48.4%)	68 (82.9%)	<0.001
Balloon-expandable valves	47 (51.6%)	14 (17.1%)	<0.001
Stent size, median	26 (26–29)	29 (26–29)	<0.001
Valve-in-valve	3 (3.3%)	0 (0%)	0.098
Trace—mild PVL	11 (12.1%)	27 (32.9%)	<0.001
Re-intubation	10 (11.1%)	6 (7.3%)	0.392
**New conduction disturbances and arrhythmias**	6 (6.6%)	9 (11.0%)	0.306
**Neurologic events**	5 (5.5%)	0 (0%)	0.061
**Myocardial infarctions**	0 (0%)	1 (1.2%)	0.233
Rethoracotomy	6 (6.6%)	12 (14.6%)	0.084
Bleeding	5 (5.5%)	4 (4.8%)	1.000
Wound healing disorder	1 (1.1%)	6 (7.3%)	0.054
Pacemaker implantation	9 (9.9%)	10 (12.2%)	0.628
**Blood transfusions, units, median**	0 (0–1)	2 (0–4)	0.001
**30-day all cause mortality**	6 (6.6%)	7 (8.5%)	0.364
6-month mortality	21 (23.1)	21 (25.6)	0.698
**Rehospitalization rate**	3 (3.3%)	5 (6.1%)	0.608
**Vascular and access related complications**	1 (1.1%)	5 (6.1%)	0.062
**Cardiac structural complications**	2 (2.2%)	6 (7.3%)	0.216
**Acute kidney injury**	6 (6.6%)	4 (4.9%)	0.168
Procedure duration, minutes Mean ± SD	91 (75.25–106.75) 92.2 ± 23.1	91 (80–101.5) 97.2 ± 39.5	0.488
Length of ICU stay, days, median Mean ± SD	1 (1–4.5) 3.8 ± 5.6	2 (1–5) 4.5 ± 7.0	0.506
Length of hospitalization, days, median Mean ± SD	9 (8–13) 11.6 ± 6.1	12 (8–18.25) 14.8 ± 10.7	0.011

The median has been indicated only for stent size, blood units, procedure duration and length of stay. All other parameters are given in numbers. ICU = intensive care unit; PVL = paravalvular leakage; SD = standard deviation. VARC criteria are presented in bold.

**Table 4:** Multifactorial Cox regression analysis of factors affecting mortality.

Factors	HR	HR 95% CI	*p* Value
Re-intubation	2.586	1.350–4.954	0.004
Acute kidney injury	6.585	2.908–14.912	<0.001

## Data Availability

The data presented in this study are available on request from the corresponding author. The data are not publicly available due to privacy reasons.

## Abbreviations

AV	Aortic Valve
COPD	chronic obstructive pulmonary disease
CT	computed tomography
ICU	intensive care units
LVEF	left ventricular ejection fraction
NYHA	New York Heart Association
PCI	percutaneous coronary intervention
PVL	paravalvular leakage
SD	standard deviation
STS PROM	The Society of Thoracic Surgeons Predicted Risk of Mortality
TAVI	Transcatheter aortic valve implantation
TA-TAVI	Transapical TAVI
TAO-TAVI	Transaortic TAVI via an upper mini-sternotomy
TF-TAVI	Transfemoral TAVI
VARC3	Valve Academic Research Consortium 3
